# Metachronous gastric metastasis from lung primary, with synchronous pancreatic neuroendocrine carcinoma

**DOI:** 10.1002/ccr3.1571

**Published:** 2018-05-08

**Authors:** Ihab I. El Hajj, Karen A. Lawrence, Temel Tirkes, Safi Shahda, Stuart Sherman

**Affiliations:** ^1^ Division of Gastroenterology Indiana University School of Medicine Indianapolis IN USA; ^2^ Division of Gastroenterology Saint George Hospital University Medical Center University of Balamand Beirut Lebanon; ^3^ Department of Pathology and Laboratory Medicine Indiana University School of Medicine Indianapolis IN USA; ^4^ Department of Radiology Indiana University School of Medicine Indianapolis IN USA; ^5^ Division of Hematology/Oncology Indiana University School of Medicine Indianapolis IN USA

**Keywords:** endoscopic ultrasound, gastric metastasis, lung cancer, neuroendocrine carcinoma, pancreatic cancer

## Abstract

The finding of gastric metachronous metastasis, several years after the diagnosis of primary lung large cell carcinoma is rare and incidental. Even more extremely rare is the finding of a synchronous primary pancreas cancer. EUS‐FNA with immunohistochemistry is useful for diagnosing metastatic lesions and differentiating those from synchronous primary lesions.

## 1 CASE DESCRIPTION

A 59‐year‐old man presented with eight‐week history of nausea, abdominal pain, and 20‐lbs weight loss. Two years prior to the current presentation, the patient was diagnosed with a stage T1A nonsmall cell lung cancer of the right upper lobe (large cell carcinoma) and his initial staging Positron Emission Tomography and Computed Tomography (PET‐CT) was negative except for the above‐mentioned lesion. The patient refused surgery at the time and completed Stereotactic Body Radiation Therapy (SBRT). His serial six‐month interval surveillance imaging has been negative.

**Figure 1:**
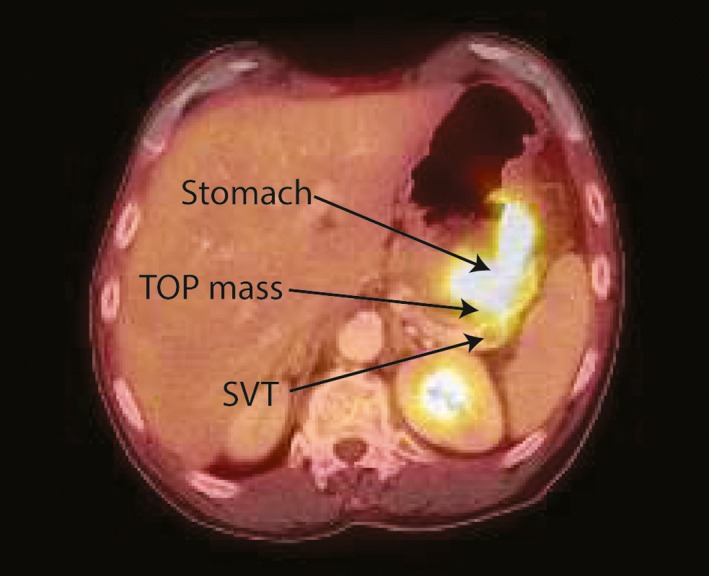
PET‐CT image showing thickening of the wall of the gastric fundus, a mass in the tail of the pancreas (both with increased radiotracer uptake), and a 1.1‐cm filling defect within the splenic vein

**Figure 2:**
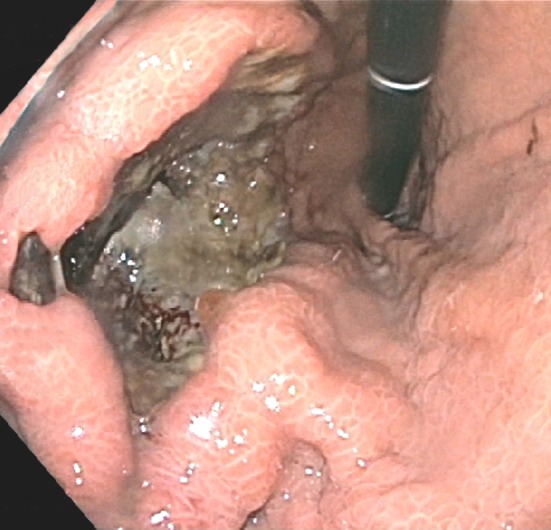
Endoscopy image showing a 5‐cm infiltrative and ulcerated mass with heaped‐up margins and necrotic center in the gastric fundus

**Figure 3:**
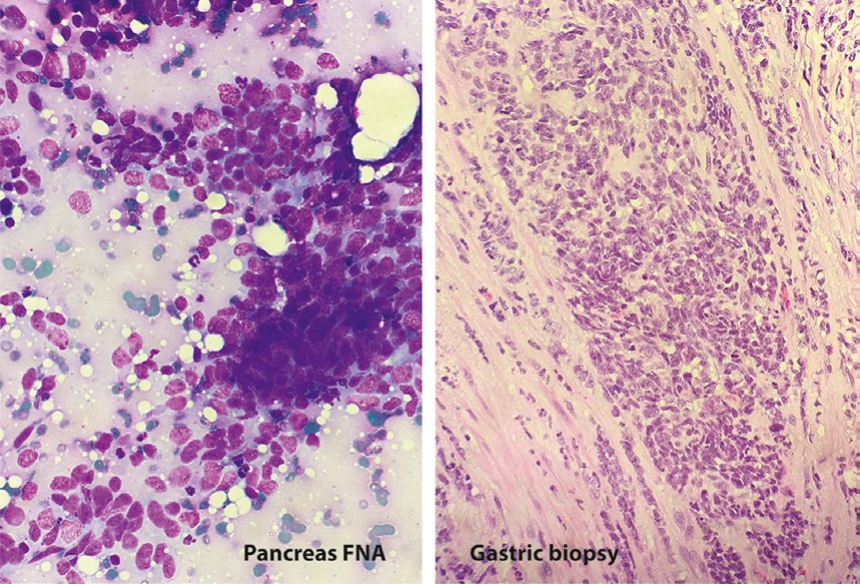
Fine needle aspirate (FNA) of the tail of pancreas mass exhibits loosely cohesive groups of cells with high nuclear/cytoplasmic ratios and nuclear molding (Diff Quik, x40). Gastric biopsy exhibits sheets of closely packed cells with vesicular nuclei and scant to absent cytoplasm (H&E, x20)

**Figure 4:**
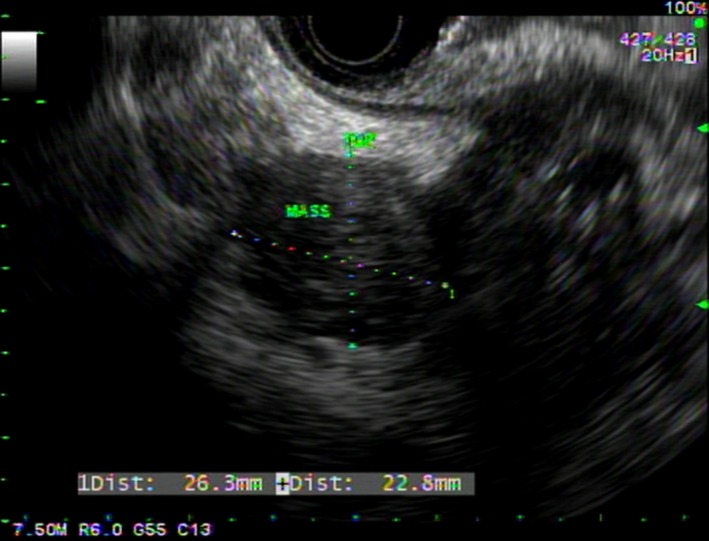
EUS image showing a 26 mm × 23 mm hypoechoic round mass in the tail of the pancreas with local vascular involvement and splenic vein thrombus

With his prior history of cancer and the current presentation, a total body PET‐CT was ordered. This showed thickening of the wall of the gastric fundus, a mass in the tail of the pancreas (both with increased radiotracer uptake), and a 1.1‐cm filling defect within the splenic vein consistent with thrombus (Figure 1). Upper endoscopy showed a 5‐cm infiltrative and ulcerated mass with heaped‐up margins and necrotic center located in the gastric fundus (Figure 2). Gastric biopsies showed poorly differentiated carcinoma (positive TTF1 and cytokeratin AE1‐3, negative CDX‐2) (Figure 3), suggestive of metachronous metastasis from a lung primary. Endoscopic ultrasound (EUS) was performed and showed a 26 mm × 23 mm hypoechoic round mass in the tail of the pancreas with local vascular involvement and splenic vein thrombus (Figure 4). EUS‐guided fine needle aspiration (FNA) and fine needle biopsy (FNB) of the mass was performed through the gastric lumen (away from the gastric tumor to avoid tumor contamination or seeding by the needle tract). This confirmed neuroendocrine carcinoma (positive synaptophysin and chromogranin, positive Ki‐67 and CDX‐2, negative TTF1) (Figure 3). Findings were suggestive of synchronous primary pancreatic neuroendocrine carcinoma. Treatment options were discussed with the patient and his family. Patient opted for palliative care and received symptomatic treatment.

Metastasis of primary lung tumor to the stomach is infrequent and sporadic.^1^ The pathogenesis is thought to be related to the tumor cell spread via the hematogenous and lymphatic routes, but there is no specific data demonstrating a particular tropism for a segment of the gastrointestinal (GI) tract.^1^ Only 21 sporadic cases have been reported in the English literature and involved different malignant cell types: squamous cell carcinoma (10 cases), adenocarcinoma (6 cases), small cell carcinoma (2 cases), pleomorphic (2 cases), and large cell carcinoma (1 case).^2^ Symptomatic cases presented with epigastric pain, chronic anemia, signs of GI bleed (hematemesis, melena), and gastric perforations (in two cases). The definite role of PET‐CT in the diagnosis of GI metastasis from lung cancer is still controversial because of the few cases and lack of enough clinical data.^1^ EUS‐FNA in combination with immunohistochemistry is useful for diagnosing metastatic lesions and differentiating those from synchronous primary lesions.^3^, ^4^

## CONFLICT OF INTEREST

None declared.

## CONSENT

Informed consent was obtained for this case report.

## AUTHORSHIP

IE and SS: involved in management of the patient. TT: reviewed the radiology images. KL: reviewed the cyotology and pathology slides. All authors were involved in revision of the manuscript and approval of the final draft.
